# CRP-Induced NLRP3 Inflammasome Activation Increases LDL Transcytosis Across Endothelial Cells

**DOI:** 10.3389/fphar.2019.00040

**Published:** 2019-01-30

**Authors:** Fang Bian, Xiao-Yan Yang, Gao Xu, Tao Zheng, Si Jin

**Affiliations:** ^1^Department of Endocrinology, Institute of Geriatric Medicine, Liyuan Hospital, Tongji Medical College, Huazhong University of Science and Technology, Wuhan, China; ^2^Department of Pharmacy, Xiangyang Central Hospital, Affiliated Hospital of Hubei University of Arts and Science, Xiangyang, China; ^3^Department of Pharmacology, Hubei Key Laboratory of Drug Target Research and Pharmacodynamic Evaluation, School of Basic Medicine, Tongji Medical College, Huazhong University of Science and Technology, Wuhan, China; ^4^Department of Pharmacy, Wuhan Union Hospital, Tongji Medical College, Huazhong University of Science and Technology, Wuhan, China; ^5^Department of Pharmacy, Taihe Hospital, Hubei University of Medicine, Shiyan, China

**Keywords:** NLRP3 inflammasome, C-reactive protein, endothelial cells, low density lipoprotein, transcytosis

## Abstract

The NLRP3 inflammasome, a multiprotein cytosolic complex that activates the IL-1 family of cytokines, plays an important role in atherosclerosis (AS). High-sensitivity c-reactive protein (hs-CRP) is widely recognized as a major cardiovascular risk predictor and recent studies name NLRP3 as a predictor of CRP levels. Mounting evidence has indicated that subendothelial retention of apolipoprotein B100-containing lipoproteins, such as low-density lipoprotein (LDL), is the initial step of atherogenesis, and is usually termed the “response to retention hypothesis.” We previously reported that CRP promotes AS by directly increasing LDL transcytosis across endothelial cells (ECs). The present study aims to investigate the effects of CRP on NLRP3 inflammasome activation and the role of the NLRP3 inflammasome in CRP-induced LDL transcytosis. We found that CRP upregulated NF-κB activity, the NF-κB inhibitor (BAY-11-7082) and Fcγ receptors (FcγRs) inhibitor (CD32/64Ab) blocked CRP-induced NF-κB activation. CRP also induced expression of pro-IL-1β and NLRP3, while BAY and CD32/64 Ab suppressed CRP-mediated expression of NLRP3 and pro-IL-1β. Moreover, CRP activated the NLRP3 inflammasome in ECs. NADPH oxidase inhibitor, diphenylene iodonium (DPI) and dithiothreitol (DTT), a broad-spectrum P2 receptor inhibitor, oxidized ATP (oATP), and a broad inhibitor of cysteine proteases, E-64d, inhibited CRP-induced NLRP3 inflammasome activation. Furthermore, NLRP3 siRNA and caspase-1 inhibitor blocked CRP-mediated LDL transcytosis across ECs. In conclusion, NLRP3 inflammasome activation was shown to be involved in CRP-mediated LDL transcytosis across ECs. CRP not only increased the expression of pro-IL-1β and NLRP3 via the FcγRs/NF-κB pathway, but also promoted NLRP3 inflammasome activation and IL-1β maturation by upregulation of reactive oxygen species (ROS) levels, purinergic receptor signaling, and activation of cysteine proteases.

## Introduction

Recent studies have suggested low-grade systemic inflammation is closely related with metabolic syndrome. Low-grade chronic inflammation is characterized by a 2-3-fold increase in the systemic concentrations of cytokines including TNF-α, IL-6, and C-reactive protein (CRP). Excessive levels of proinflammatory cytokines may have facilitate the shift of the endothelial cell (EC) phenotype from a quiescent to an activated phenotype.

Although controversies regarding CRP as a causal agent for atherothrombosis remain, no study has denied that CRP serves as a biomarker for cardiovascular events. Many reports have suggested that high level of CRP is associated with the increased risk of atherosclerosis (AS). Results from justification for the use of statins in prevention: an intervention trial evaluating rosuvastatin (JUPITER) and a meta-analysis from primary prevention trials demonstrated that rosuvastatin reduces cardiovascular disease (CVD) events in women with elevated hs-CRP or dyslipidemia (Mora et al., 2010). Phospholipase A2-dependent dissociation of circulating pentameric CRP (pCRP) to monomeric CRP (mCRP) localized and aggravated inflammation in AS and myocardial infarction (Braig et al., 2017). 1,6-bis(phosphocholine)-hexane inhibited the proinflammatory activity of mCRP by stabilizing pCRP (Braig et al., 2017). Mounting evidence has indicated that subendothelial retention of apolipoprotein B100-containing lipoproteins, such as low-density lipoprotein (LDL), is the initial step of atherogenesis, and is usually termed the “response to retention hypothesis.” (Williams and Tabas, 1995). Zwaka et al. (2001) investigated that foam cell formation in human atherogenesis may be caused in part by CRP-opsonized native LDL. In a previous study, we found that CRP accelerates AS by directly increasing LDL transcytosis across ECs (Bian et al., 2014).

IL-1β is a key cytokine that plays an important role in the initiation and development of atherogenesis. Deficiency of IL-1β in apoE^-/-^ mice reduced the severity of AS (Isoda et al., 2004). Chronic treatment with IL-1β promoted the formation of coronary intimal lesions in cholesterol-fed pigs (Shimokawa et al., 1996). Pro-IL-1β (p31) is cleaved to form active IL-1β (p17) by caspase-1, a process that is tightly regulated by inflammasomes. The NLRP3 inflammasome is a caspase-1 activating platform, and contains the sensor NLRP3, the adapter apoptosis associated speck-like protein containing a CARD (ASC), and the effector caspase-1. Collaboration between epidemiologic, clinical, and bench investigators has now led to randomized outcome trials targeting the CRP/IL-6/IL-1β axis (Ridker, 2016). Moving upstream from CRP in the inflammatory cascade provided novel targets for atheroprotection that focus on the central IL-6 signaling pathway and ultimately on inhibition of the NLRP3 inflammasome (Ridker, 2016).

Converging lines of evidence have shown that multiple AS risk factors can activate the NLRP3 inflammasome. Cholesterol crystals in atherosclerotic lesions, recognized as an important hallmark of AS, can trigger the NLRP3 inflammasome and induce a robust release of IL-1β in monocytes, which is linked to the release of lysosomal protease (Duewell et al., 2010; Rajamäki et al., 2010). Furthermore, cholesterol crystals induced activation of neutrophils triggers the release of neutrophil extracellular traps (NETs), which can prime macrophages to produce pro-IL-1β (Warnatsch et al., 2015). NETs are extracellularly released DNA fibers that form “netlike” structures, which bind bacteria and platelets and exert multiple cytotoxic effects. In the process of “NETosis,” neutrophils expele cytosolic and nuclear material, which lead to acute thrombosis and is associated with several pro-atherosclerotic processes (Nahrendorf and Swirski, 2015). It was also reported that cholesterol crystal induce activation of the NLRP3 inflammasome in a study examining the contribution of alcohol consumption to the early development of AS (Abdul-Muneer et al., 2017). In addition, minimally modified LDL (mmLDL) can activate the NF-κB pathway, lead to lysosome destabilization, and subsequently induce NLRP3 inflammasome activation (Duewell et al., 2010). McGeough et al. (2017) demonstrated that TNF-α plays an important role in promoting NLRP3 inflammasomopathies in cryopyrin-associated periodic syndromes (CAPS).

Early studies have reported that CRP can increase the production of cytokines including IL-1β and IL-1α (Galvede et al., 1993; Pue et al., 1996). Meta-analysis of genome-wide association studies has identified NLRP3 as a predictor of CRP levels (Dehghan et al., 2011). A recent study revealed that rosmarinic acid inhibits nicotine-induced AS by suppressing the ROS-NLRP3 inflammasome-CRP axial (Yao et al., 2018). These reports suggest a link between increased levels of CRP and the NLRP3 inflammasome, and thus, a link to AS. However, whether or not the NLRP3 inflammasome can promote LDL transport across ECs and accelerate lipid deposition in vessels, is not clear. In the present study, the specific roles of NLRP3 inflammasome activation in CRP-induced LDL transcytosis across ECs were investigated.

## Materials and Methods

### Isolation and Culture of HUVECs

The collection of human umbilical cords was approved by the Ethics Committee of Tongji Medical College, Huazhong University of Science and Technology (Wuhan, China), and conducted in accordance with the Declaration of Helsinki. Primary HUVECs were isolated from newborn umbilical cords as described previously (Bian et al., 2014). HUVECs were cultured in the endothelial cell medium (ECM; ScienCell) with 10% FBS, 100 U/mL penicillin, 100 U/mL streptomycin (ScienCell) and 30 μg/mL endothelial cell growth supplement (ECGS; ScienCell) at 37°C with 5% CO_2_. Cells were passaged when 80∼90% confluent and were used between passages 2 and 9.

### LDL Labeling

Low-density lipoprotein was labeled using fluorescein isothiocyanate (FITC; Biosharp) as described previously (Bian et al., 2014). All procedures were performed in the dark. 2 mg LDL (The institute of Clinical Pharmacology of Sun Yat-sen University) and 120 μg FITC were mixed and incubated at 37°C for 2 h. Unbound FITC was removed by dialysis against PBS for 72 h at 4°C. After the measurement of protein with the BCA assay kit (Thermo Scientific), FITC-LDL was stored at 4°C for further use.

### NF-κB Activity Assay

HUVECs were pretreated with 1 μmol/L Bay-11-7082 (BAY; Cayman Chemical) for 1 h, or 10 μg/mL CD32Ab (Santa Cruz) or 10 μg/mL CD64Ab (Santa Cruz) for 2 h, followed by 20 μg/mL CRP (Sigma) stimulation for 24 h. The NF-κB activity was measured according to an ELISA-based method described previously (Jin et al., 2005).

### siRNA Transfection

HUVECs were transfected with NLRP3 siRNA (Guangzhou RiboBio, China) using Hiperfect Transfection Reagent (QIAGEN) according to manufacturer’s instruction. Non-specific Scrambled siRNA (Guangzhou RiboBio, China) was transfected as control. Briefly, HUVECs at passage 2/3 were seeded in 6-well plates (Costar) or on polyester membranes (Costar transwell, 6.5 mm diameter, 0.4 μm pore size). When cells reached 30–50% confluency, the cells were incubated with the transfection mix at 37°C for 24 h. The medium was changed 24 h later. The level of protein expression of NLRP3, pro-caspase-1, caspase-1, pro-IL-1β, IL-1β, and LDL transcytosis were evaluated after 24 h.

### Western Blotting Analysis

Cells were lysed with the RIPA lysis buffer (Beyotime Institute of Biotechnology) containing protease inhibitors. The proteins were separated by the SDS-PAGE gel and transferred to a PVDF membrane. The membranes were probed with primary antibodies against NLRP3 (1:500; Proteintech), IL-1β (1:600; Cell Signaling), Caspase-1 (1:800; Cell Signaling), and β-actin (1:4000; Proteintech). The immune-reactive bands were visualized by the ECL (Thermo Scientific) Western blot detection system.

### LDL Transcytosis

As described in our previous studies (Bian et al., 2014), the amount of LDL transcytosis was measured by a non-radioactive method *in vitro.* In brief, HUVECs were seeded on a polyester membrane (∼4 × 10^4^ cells/insert). The integrity of cell monolayer was determined by a method described previously (Cankova et al., 2011) which simply refers to fill the upper chamber to the top and then leave the cells overnight and the fall in the fluid level in the top chamber was measured to reflect the leak. Culture inserts with equal leak were selected to conduct the assay. Two inserts of cell monolayers with equal integrity were divided into the same group: the non-competitive insert and the competitive insert. The non-competitive insert was incubated with FITC-LDL (50 μg/mL) for 24 h to determine the total amount of transendothelial LDL. Paracellular transport was determined by incubating the cells with FITC-LDL (50 μg/mL) and 6-fold excess of unlabeled LDL in the competitive insert. The FITC fluorescent intensity was measured via a fluorescence spectrophotometer (TECAN, INFINITE F200PRO) with excitation and emission wavelengths of 490 and 520 nm, respectively. Background fluorescence was determined by measuring the serum-free ECM. As a matter of fact, the amount of LDL transcytosis is the difference in FITC fluorescent intensity between the non-competitive insert and the competitive insert. HUVECs were pretreated with 20 μmol/L Z-VAD-FMK for 1 h, Scrambled siRNA, or NLRP3 siRNA for 24 h, followed by incubating with CRP and FITC-LDL (50 μg /mL) for another 24 h.

### Statistical Analysis

All data were expressed as the mean ± SEM from at least three separate experiments. Individual group statistical comparisons were analyzed by unpaired Student *t*-test; and multiple-group comparisons were evaluated by one-way ANOVA with *post hoc* testing; A value of *P* < 0.05 was considered statistically significant.

## Results

### CRP Activates the NLRP3 Inflammasome

**Figure 1:**
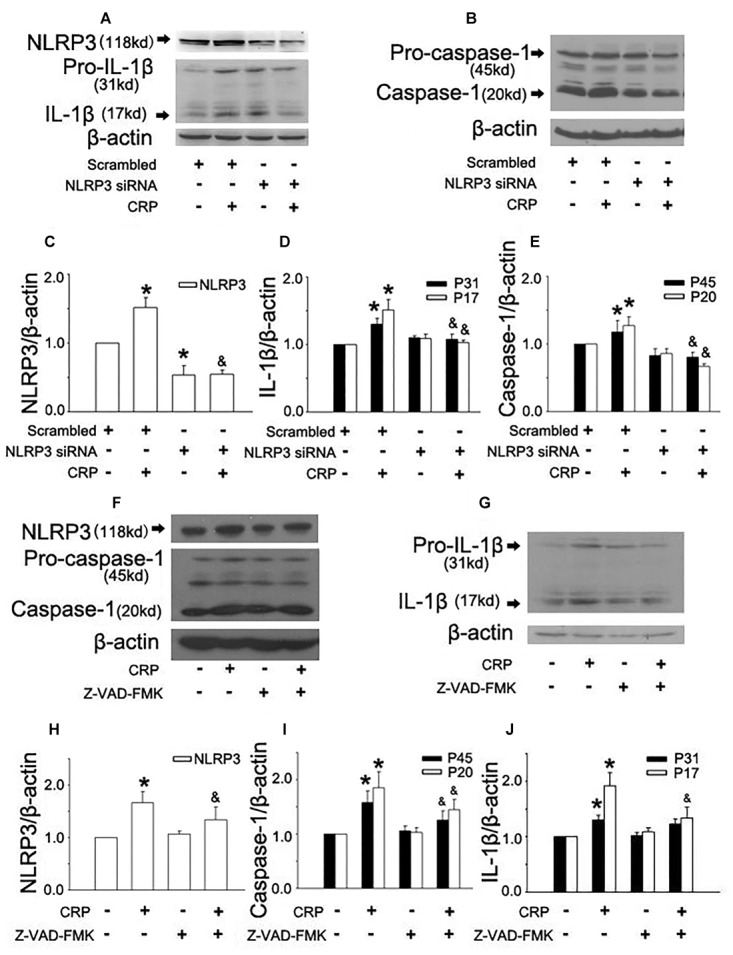
Effect of C-reactive protein (CRP) on NLRP3 inflammasome activation. HUVECs were transfected with 20 μmol/L NLRP3 siRNA, 20 μmol/L Scrarmbled siRNA for 24 h **(A–E)** or incubated with 20 μmol/L Z-VAD-FMK for 1 h **(F–J)**, and then were treated in the absence or presence of 20 μg/mL CRP for 24 h. **(A**,**B**,**F**,**G)** Representative western blots showing the expression of NLRP3, pro-caspase-1, caspase-1, pro-IL-1β, and IL-1β. **(C–E**,**H–J)** Summary bar graphs showing the expression of proteins. ^∗^*P* < 0.05 vs. Scrambled siRNA or Ctr; ^&^*P* < 0.05 vs. Scrambled siRNA+CRP or CRP; *n* = 4.

In the study, we first studied the effect of CRP on NLRP3 inflammasome activation in ECs. As summarized in Figure 1, the expression of proteins involved in the NLRP3 inflammasome signaling pathway (NLRP3, pro-caspase-1, and pro-IL-1β) were up-regulated after incubating with CRP for 24 h. CRP also induced the activation of NLRP3, with the ensuing increased caspase-1 and IL-1β. HUVECs were transfected with NLRP3 siRNA to specifically knock down NLRP3 expression, which blunted the effects of CRP-induced NLRP3, pro-caspase-1/caspase-1, and pro-IL-1β/IL-1β expression (Figure 1A–E). These results suggested that CRP provides the signals for the production of NLRP3 and pro-IL-1β and NLRP3 inflammasome activation.

Inflammasome activation results in the recruitment and activation of caspase-1, the key enzyme involed in the processing of pro-IL-1β into mature IL-1β. To confirm the role of caspase-1 in CRP-induced IL-1β production, caspase-1 was inhibited by pre-treatment Z-VAD-FMK for 1 h before CRP exposure. Z-VAD-FMK diminished the expression of all proteins except for pro-IL-1β as depicted in Figure 1F–J.

### CRP Induces the Expression of NLRP3 and Pro-IL-1β Through FcγRs and NF-κB Activation

**Figure 2:**
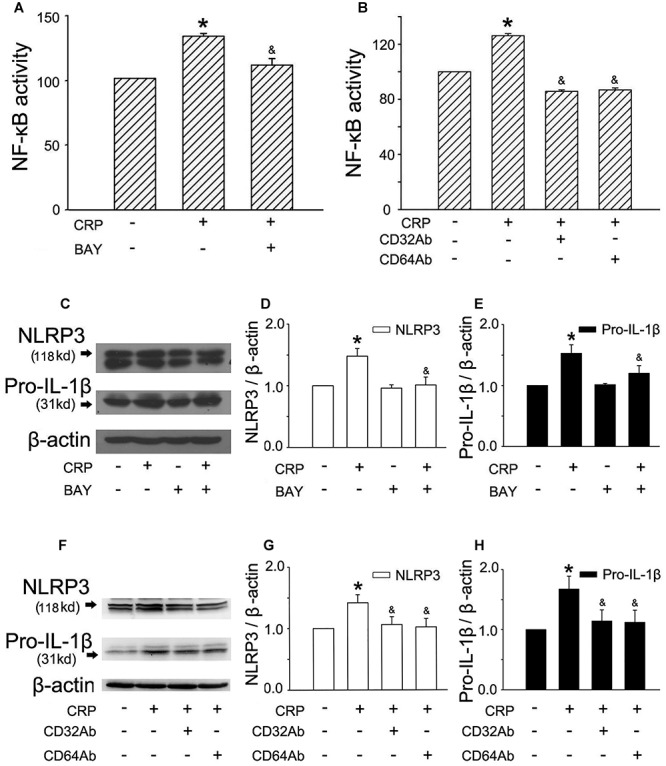
FcγRs and NF-κB are involved in CRP-mediated NLRP3 and pro-IL-1β expression. HUVECs were incubated with 1 μmol/L BAY for 1 h, and then were treated in the absence or presence of 20 μg/mL CRP for 24 h **(A,C)**. HUVECs were incubated with 10 μg/mL CD32Ab, or 10 μg/mL CD64Ab for 2 h, and then were treated in the absence or presence of 20 μg/mL CRP for 24 h **(B,F)**. **(A,B)** NF-κB activity was assayed. **(C,F)** Representative western blots showing the expression of NLRP3 and pro-IL-1β. **(D**,**E**,**G**,**H)** Summary bar graphs showing the expression of proteins. ^∗^*P* < 0.05 vs. Ctr; ^&^*P* < 0.05 vs. CRP; *n* = 4.

It is widely accepted that an initial priming signal is required (referred as signal 1) to induce the expression of pro-IL-1β and NLRP3, which is tightly associated with NF-κB activation (Hoseini et al., 2017). To elucidate the mechanism of CRP-mediated NLRP3 and pro-IL-1β expression, we stimulated cells with a NF-κB inhibitor, BAY-11-7082 (BAY), and CD32/CD64 antibody (CD32Ab/CD64Ab). We evaluated NF-κB activity using an ELISA-based transcriptional factor-DNA binding activity assay (Jin et al., 2005). As summarized in Figure 2A, CRP was able to upregulate NF-κB activity, while BAY blocked CRP-induced NF-κB activation. Furthermore, BAY suppressed the CRP-mediated increase in NLRP3 (Figure 2C,D) and pro-IL-1β (Figure 2C,E) expression.

C-reactive protein has been shown to interact with plasma membrane-associated FcγRs. As reported in studies, CRP reduced eNOS activity and increased endothelial cell-monocyte adhesion expression through FcγRII (CD32) and FcγRI (CD64) in ECs (Tanigaki et al., 2015). To explore the specific receptors interacting with CRP involved in our study, CD32 antibody (CD32Ab), and CD64 antibody (CD64Ab) were used. As shown in Figure 2B, either CD32Ab or CD64Ab disturbed CRP-induced NF-κB activation. Moreover, CD32Ab or CD64Ab also blocked CRP-mediated NLRP3 (Figure 2F,G) and pro-IL-1β (Figure 2F,H) expression.

### Three Mechanisms Are Involved in CRP-Induced NLRP3 Inflammasome Activation

The second step (named as signal 2) is involved in maturation of IL-1β via activation of the NLRP3 inflammasome. We further explored the underlying mechanisms by which CRP activated the NLRP3 inflammasome. During the processing for NLRP3 inflammasome activation, at least one of following three events is required: the increased production of intracellular reactive oxygen species (ROS) production, purinergic receptor signaling and activation of cysteine proteases.

**Figure 3:**
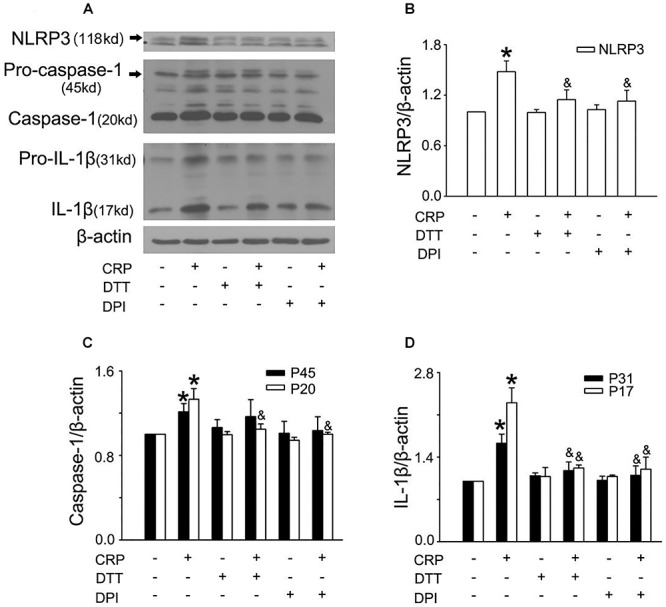
Reactive oxygen species (ROS) are implicated in CRP-induced NLRP3 inflammasome activation. HUVECs were incubated with 10 μmol/L DPI, or 30 μmol/L DTT for 30 min, and then were treated in the absence or presence of 20 μg/mL CRP for 24 h. **(A)** Representative Western blots showing the expression of NLRP3, pro-caspase-1, caspase-1, pro-IL-1β, and IL-1β. **(B–D)** Summary bar graphs showing the expression of proteins.^∗^*P* < 0.05 vs. Ctr; ^&^*P* < 0.05 vs. CRP; *n* = 4.

**Figure 4:**
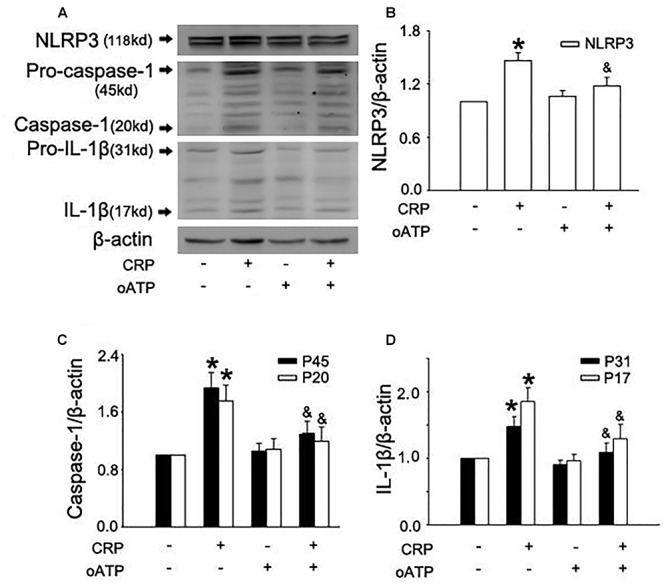
Purinergic receptor signaling is implicated in CRP-induced NLRP3 inflammasome activation. HUVECs were incubated with 100 μmol/L oATP for 1 h, and then were treated in the absence or presence of 20 μg/mL CRP for 24 h. **(A)** Representative Western blots showing the expression of NLRP3, pro-caspase-1, caspase-1, pro-IL-1β, and IL-1β. **(B–D)** Summary bar graphs showing the expression of proteins. ^∗^*P* < 0.05 vs. Ctr; ^&^*P* < 0.05 vs. CRP; *n* = 4.

**Figure 5:**
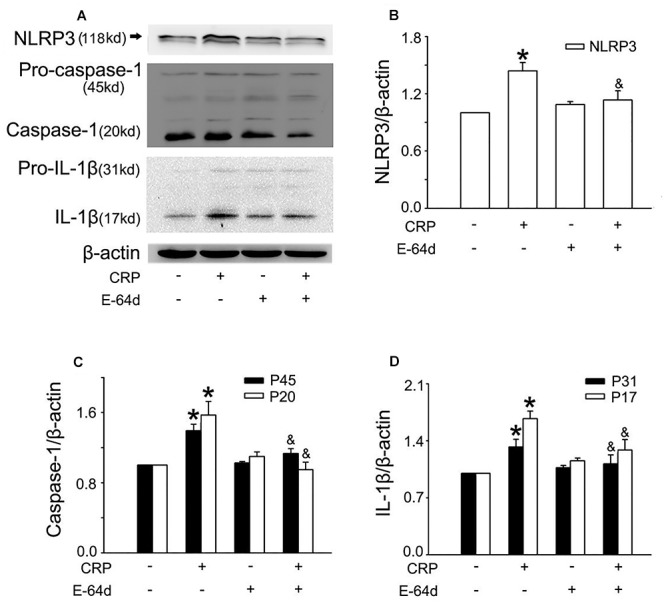
Cysteine proteases are associated with CRP-induced NLRP3 inflammasome activation. HUVECs were incubated with 50 μmol/L E-64d for 1 h, and then were treated in the absence or presence of 20 μg/mL CRP for 24 h. **(A)** Representative Western blots showing the expression of NLRP3, pro-caspase-1, caspase-1, pro-IL-1β, and IL-1β. **(B–D)** Summary bar graphs showing the expression of proteins. ^∗^*P* < 0.05 vs. Ctr; ^&^*P* < 0.05 vs. CRP; *n* = 4.

In a previous study, we found that CRP increases ROS levels in HUVECs (Bian et al., 2014). To further clarify the role of ROS in CRP-induced NLRP3 inflammasome activation, we pretreated cells with an NADPH oxidase inhibitor, DPI or with the reducing agent, DTT. As summarized in Figure 3A–D, CRP-stimulated HUVECs increased levels of NLRP3, caspase-1, pro-IL-1β and IL-1β protein levels, with DPI or DTT pre-treatment prior to CRP treatment reducing the levels of all of these markers. However, they showed no effect on pro-caspase-1 expression. Previous studies have suggested ATP is involved in NLRP3 inflammasome activation and IL-1β production through multiple purinergic receptor signaling (Riteau et al., 2012). To study whether CRP-induced NLRP3 inflammasome activation is mediated via purinergic receptors, we treated cells with a broad-spectrum P2 receptor inhibitor, oATP. As shown in Figure 4A–D, CRP-induced caspase-1 activation and maturation of IL-1β was attenuated by oATP. In addition, cysteine proteases, such as cathepsins B and L, could trigger NLRP3 inflammasome activation (Duewell et al., 2010). In the present study, CRP-induced caspase-1 activation and IL-1β mature was blocked by E-64d, a broad inhibitor of cysteine proteases (Figure 5A–D).

### The NLRP3 Inflammasome Is Implicated in CRP-Mediated LDL Transcytosis Across ECs

**Figure 6:**
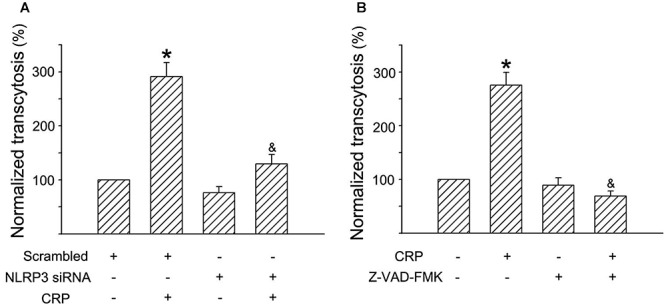
Effects of NLRP3 inflammasome on CRP-mediated LDL transcytosis in HUVECs. HUVECs were transfected with 20 μmol/L NLRP3 siRNA, 20 μmol/L Scrambled siRNA for 24 h or incubated with 20 μmol/L Z-VAD-FMK for 1 h, and then were treated in the absence or presence of 20 μg/mL CRP for 24 h. **(A)** Effects of NLRP3 siRNA on CRP-induced LDL transcytosis in HUVECs. ^∗^*P* < 0.05 vs. Scrambled siRNA; ^&^*P* < 0.05 vs. CRP; *n* = 4. **(B)** Effects of Z-VAD-FMK on CRP-induced LDL transcytosis in HUVECs. ^∗^*P* < 0.05 vs. Ctr; ^&^*P* < 0.05 vs. CRP; *n* = 4.

In a previous study, we have established an *in vitro* model of LDL transcytosis and found that CRP promotes LDL transcytosis across ECs (Bian et al., 2014). In the present study, based on the model, we further explored the effects of the NLRP3 inflammasome on CRP-induced LDL transcytosis. As described in Figure 6, CRP promoted LDL transcytosis across ECs after exposure for 24 h. NLRP3 siRNA and Z-VAD-FMK decreased CRP-induced LDL transcytosis (Figure 6A,B).

## Discussion

C-reactive protein is an acute phase protein with pleiotropic cytokine-like properties. We previously reported that CRP promotes AS by directly increasing the transcytosis of LDL across ECs (Bian et al., 2014). In the present study, we found that CRP is also a potent activator of the NLRP3 inflammasome. Moreover, either silencing NLRP3 or inhibiting caspase-1 activity attenuated CRP-induced LDL transcytosis across ECs, suggesting a NLRP3 inflammasome-mediated mechanism in CRP-induced LDL transcytosis.

IL-1β, a major atheroprone factor, is activated via a two-step process. An initial priming step (signal 1) induces the expression of pro-IL-1β and NLR proteins (e.g., NLRP3), which is tightly associated with NF-κB signaling. The second step (signal 2) stimulates the assembly and activation of inflammasomes to regulate IL-1β maturation and secretion. In this study, we demonstrated that CRP could provide signals to induce expression of pro-IL-1β and NLRP3, activate the NLRP3 inflammasome and promote IL-1β maturation. Using the caspase-1 inhibitor Z-VAD-FMK, we showed that IL-1β maturation induced by CRP is dependent on caspase-1, suggesting the involvement of an inflammasome-mediated pathway. Silencing NLRP3, abolished CRP-induced IL-1β expression, further implicating an inflammasome- IL-1β axis-mediated mechanism and identifying NLRP3 inflammasome as the CRP-responsive element in this process.

Minimally modified LDL is able to activate the NF-κB pathway, lead to lysosome destabilization, and subsequently induce NLRP3 inflammasome activation (Duewell et al., 2010). Serum amyloid A can act as signal 1 to activate the NF-κB pathway and as signal 2 to induce NLRP3 inflammasome activation via purinergic ligand-gated ion channel 7 receptor (P2X7R) and the cathepsin B-sensitive pathway (Niemi et al., 2011). TNF-α upregulates NF-κB activity to increase pro-IL-1β expression and ROS levels in SK-N-MC cells. ROS are sensed by NLRP3 and mediate NLRP3 inflammasome activation leading to the release of IL-1β (Álvarez and Muñoz-Fernández, 2013). Similarly, in the present study, CRP also served as both signal 1 and signal 2 for NLRP3 inflammasome activation. To this extent, we further explored the underlying mechanism for CRP-induced NLRP3 inflammasome activation.

An NF-κB-dependent priming signal contributes to the production of pro-IL-1β and NLRP3, which may be a rate-limiting component of inflammasome complex formation. In our case, we found that CRP upregulates the activity of NF-κB and BAY blocked CRP-induced expression of pro-IL-1β and NLRP3. It was suggested that increased NF-κB activity is involved in the priming signal provided by CRP. Emerging evidence revealed that CRP largely enhances expression of the adhesion molecules (VCAM-1 and ICAM-1), and inhibits production of eNOS and prostacyclin, accelerating the progression of AS via the FcγRs (CD32 and CD64) in cultured human aortic endothelial cells (HAECs) (Devaraj et al., 2005; Devaraj et al., 2006). In addition, previous analysis also indicated that CRP activates the NF-κB pathway, induces VCAM-1 expression through CD32 on HUVECs or HAECs, and promotes EC-monocyte interactions, and thus facilitates the development of AS (Liang et al., 2006). In the present study, the antibodies against CD32 or CD64 suppressed CRP-induced NF-κB activation and the expression of pro-IL-1β and NLRP3. Our results indicated that CRP-mediated pro-IL-1β and NLRP3 expression is regulated by the FcγR/NF-κB pathway.

During the processing for inflammasome activation, at least one of following three events is required: ROS production, purinergic receptor signaling, and activation of cysteine proteases. ROS derived from NADPH oxidases or mitochondria are proposed to activate NLRP3. Several studies described that ROS can induce the dissociation of thioredoxin (TRX)-interacting protein (TXNIP) from thioredoxin and allow it to directly bind to NLRP3 (Thompson et al., 2014). We previously reported that CRP increases intracellular level of ROS, and both the NADPH oxidase inhibitor DPI and the reducing agent DTT reduced CRP-induced increased in ROS and LDL transcytosis across ECs (Bian et al., 2014). Here, we showed that DPI and DTT blocked CRP-mediated NLRP3 inflammasome activation and IL-1β maturation, indicating that ROS play a critical role in this process. ATP or its metabolites are able to signal through different purinergic receptors. Extracellular ATP increases the open probability of purinergic receptors and leads to K^+^ efflux. K^+^ efflux induces membrane pannexin-1 pore formation and activates NLRP3 inflammasome. Previous studies have suggested that ATP is involved in NLRP3 inflammasome activation and IL-1β production through multiple purinergic receptor signaling (Riteau et al., 2012). In the present study, we found that all proteins are inhibited by the broad-spectrum P2 receptor inhibitor, oATP. In addition, the accumulation of specific crystals and particulate structures can lead to lysosome rupture and the release of lysosomal proteases, resulting in NLRP3 inflammasome activation. In our study, we investigated that the broad-spectrum inhibitor cysteine proteases (E-64d), also downregulates CRP-induced NLRP3 inflammasome activation.

However, this study has a few limitations. Firstly, the direct evidence of CRP-mediated NLRP3 inflammasome activation has not been examined. It is meaningful to perform Co-IP analysis in the future to verify that CRP treatment indeed stimulates assembly of the NLRP3 inflammasome. Another limitation of this study is that a mouse model to demonstrate the role of the NLRP3 inflammasome in CRP-induced LDL transcytosis needs to be investigated in future studies.

## Conclusion

**Figure 7:**
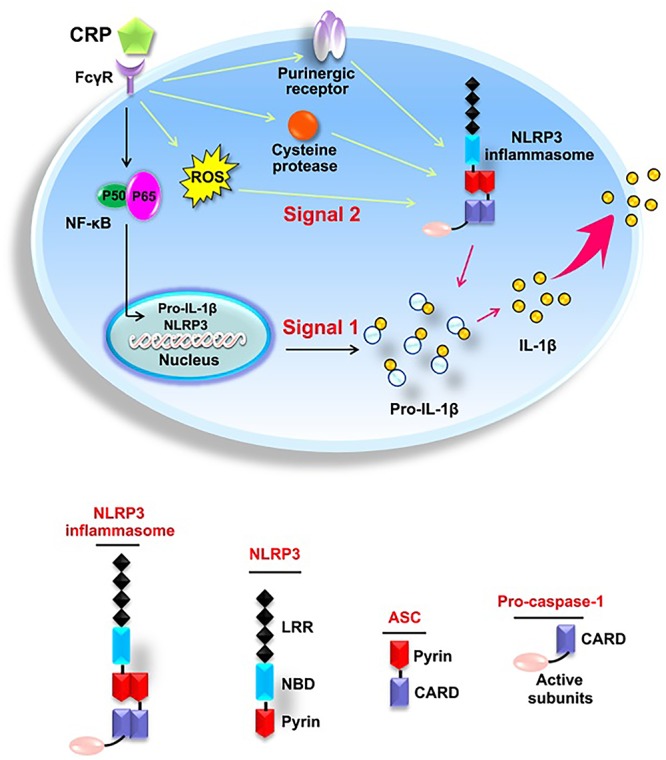
Schematic diagram of CRP-induced NLRP3 inflammasome activation.

As summarized in Figure 7, the present study elucidates the effect of CRP in mediating NLRP3 inflammasome activation. The underlying mechanisms involve the binding of CRP to CD32 and CD64, activation of NF-κB, upregulation the levels of ROS levels, purinergic receptor signaling, and increased activity of cysteine proteases. Moreover, our study indicates that the NLRP3 inflammasome is implicated in CRP-induced LDL transcytosis across ECs. These findings provide new insights into the pathogenesis of AS, as well as novel strategies for the prevention or treatment of this widespread disease.

## Author Contributions

FB and SJ conceptualized the study. FB, X-YY, and SJ contributed to formal analysis and funding acquisition. FB, GX, and TZ investigated the data. SJ supervised the data. FB, X-YY, and SJ wrote the manuscript.

## Conflict of Interest Statement

The authors declare that the research was conducted in the absence of any commercial or financial relationships that could be construed as a potential conflict of interest.

## References

[B1] Abdul-Muneer P. M. Alikunju S. Mishra V. Schuetz H. Szlachetka A. M. Burnham E. L. (2017). Activation of NLRP3 inflammasome by cholesterol crystals in alcohol consumption induces atherosclerotic lesions. *Brain Behav. Immun.* 62 291–305. 10.1016/j.bbi.2017.02.014 28232172 PMC6378699

[B2] Álvarez S. Muñoz-Fernández M. Á (2013). TNF-A may mediate inflammasome activation in the absence of bacterial infection in more than one way. *PLoS One* 8:e71477. 10.1371/journal.pone.0071477 23940760 PMC3737100

[B3] Bian F. Yang X. Zhou F. Wu P. H. Xing S. Xu G. (2014). C-reactive protein promotes atherosclerosis by increasing LDL transcytosis across endothelial cells. *Br. J. Pharmacol.* 171 2671–2684. 10.1111/bph.12616 24517733 PMC4009008

[B4] Braig D. Nero T. L. Koch H. G. Kaiser B. Wang X. Thiele J. R. (2017). Transitional changes in the CRP structure lead to the exposure of proinflammatory binding sites. *Nat. Commun.* 8:14188. 10.1038/ncomms14188 28112148 PMC5264208

[B5] Cankova Z. Huang J. D. Kruth H. S. Johnsona M. (2011). Passage of low-density lipoproteins through Bruch’s membrane and choroid ?. *Exp. Eye Res.* 93 947–955. 10.1016/j.exer.2011.10.016 22063729 PMC3242000

[B6] Dehghan A. Dupuis J. Barbalic M. Bis J. C. Eiriksdottir G. Lu C. (2011). Meta-analysis of genome-wide association studies in > 80 000 subjects identifies multiple loci for C-reactive protein levels. *Circulation* 123 731–738. 10.1161/CIRCULATIONAHA.110.948570 21300955 PMC3147232

[B7] Devaraj S. Davis B. Simon S. I. Jialal I. (2006). CRP promotes monocyte-endothelial cell adhesion via Fcgamma receptors in human aortic endothelial cells under static and shear flow conditions. *Am. J. Physiol. Heart Circ. Physiol.* 291:H1170. 10.1152/ajpheart.00150.2006 16603696

[B8] Devaraj S. Du C. T. Jialal I. (2005). Binding and internalization of C-reactive protein by Fcgamma receptors on human aortic endothelial cells mediates biological effects. *Arterioscler. Thromb. Vasc. Biol.* 25 1359–1363. 10.1161/01.ATV.0000168573.10844.ae 15860734

[B9] Duewell P. Kono H. Rayner K. J. Sirois C. M. Vladimer G. Bauernfeind F. G. (2010). NLRP3 inflammasomes are required for atherogenesis and activated by cholesterol crystals. *Nature* 464 1357–1361. 10.1038/nature08938 20428172 PMC2946640

[B10] Galvede R. B. Wiktorowicz K. Kushner I. Dayer J. M. (1993). C-reactive protein increases production of IL-1 alpha, IL-1 beta, and TNF-alpha, and expression of mRNA by human alveolar macrophages. *J. Leukoc. Biol.* 53 439–435. 10.1002/jlb.53.4.439 8482924

[B11] Hoseini Z. Sepahvand F. Rashidi B. Sahebkar A. Masoudifar A. Mirzaei H. (2017). NLRP3 inflammasome: its regulation and involvement in atherosclerosis. *J. Cell. Physiol.* 233 2116–2132. 10.1002/jcp.25930 28345767

[B12] Isoda K. Sawada S. Ishigami N. Matsuki T. Miyazaki K. Kusuhara M. (2004). Lack of interleukin-1 receptor antagonist modulates plaque composition in apolipoprotein E-deficient mice. *Arterioscler. Thromb. Vasc. Biol.* 24 1068–1073. 10.1161/01.ATV.0000127025.48140.a3 15059807

[B13] Jin S. Lu D. Ye S. Ye H. Zhu L. Feng Z. (2005). A simplified probe preparation for ELISA-based NF-kappaB activity assay. *J. Biochem. Biophys. Methods* 65 20–29. 10.1016/j.jbbm.2005.08.006 16198424

[B14] Liang Y. J. Shyu K. G. Wang B. W. Lai L. P. (2006). C-reactive protein activates thenuclear factor-κB pathway andinduces vascular cell adhesion molecule-1expression through CD32 inhuman umbilical vein endothelial cells andaortic endothelial cells. *J. Mol. Cell. Cardiol.* 40 412–420. 10.1016/j.yjmcc.2005.12.008 16430914

[B15] McGeough M. Wree A. Inzaugarat M. Haimovich A. Johnson C. Peña C. (2017). TNF regulates transcription of NLRP3 inflammasome components and inflammatory molecules in cryopyrinopathies. *J. Clin. Invest.* 127 4488–4497. 10.1172/JCI90699 29130929 PMC5707143

[B16] Mora S. Glynn R. J. Hsia J. Macfadyen J. G. Genest J. Ridker P. M. (2010). Statins for the primary prevention of cardiovascular events in women with elevated high-sensitivity C-reactive protein or dyslipidemia: results from the justification for the use of statins in prevention: an intervention trial evaluating rosuvastatin (JUPI). *Circulation* 121 1069–1077. 10.1161/CIRCULATIONAHA.109.906479 20176986 PMC4439924

[B17] Nahrendorf M. Swirski F. K. (2015). Immunology. Neutrophil-macrophage communication in inflammation and atherosclerosis. *Science* 349 237–248. 10.1126/science.aac7801 26185231

[B18] Niemi K. Teirilä L. Lappalainen J. Rajamäki K. Baumann M. H. Öörni K. (2011). Serum amyloid a activates the NLRP3 inflammasome via P2X7 receptor and a cathepsin B-sensitive pathway. *J. Immunol.* 186 6119–6128. 10.4049/jimmunol.1002843 21508263

[B19] Pue C. A. Mortensen R. F. Marsh C. B. Pope H. A. Wewers M. D. (1996). Acute phase levels of C-reactive protein enhance IL-1 beta and IL-1ra production by human blood monocytes but inhibit IL-1 beta and IL-1ra production by alveolar macrophages. *J. Immunol.* 156 1594–1600. 8568265

[B20] Rajamäki K. Lappalainen J. Öörni K. Välimäki E. Matikainen S. Kovanen P. T. (2010). Cholesterol crystals activate the NLRP3 inflammasome in human macrophages: a novel link between cholesterol metabolism and inflammation. *PLoS One* 5:e11765. 10.1371/journal.pone.0011765 20668705 PMC2909263

[B21] Ridker P. M. (2016). From C-reactive protein to interleukin-6 to interleukin-1 moving upstream to identify novel targets for atheroprotection. *Circ. Res.* 118 145–156. 10.1161/CIRCRESAHA.115.306656 26837745 PMC4793711

[B22] Riteau N. Baron L. Villeret B. Guillou N. Savigny F. Ryffel B. (2012). ATP release and purinergic signaling: a common pathway for particle-mediated inflammasome activation. *Cell Death Dis.* 3:e403. 10.1038/cddis.2012.144 23059822 PMC3481132

[B23] Shimokawa H. Ito A. Fukumoto Y. Kadokami T. Nakaike R. Sakata M. (1996). Chronic treatment with interleukin-1 beta induces coronary intimal lesions and vasospastic responses in pigs in vivo. The role of platelet-derived growth factor. *J. Clin. Invest.* 97 769–776. 10.1172/JCI118476 8609234 PMC507115

[B24] Tanigaki K. Sundgren N. Khera A. Vongpatanasin W. Mineo C. Shaul P. W. (2015). Fcγ receptors and ligands and cardiovascular disease. *Circ. Res.* 116 368–384. 10.1161/CIRCRESAHA.116.302795 25593280 PMC4331353

[B25] Thompson J. K. Westbom C. M. Macpherson M. B. Mossman B. T. Heintz N. H. Spiess P. (2014). Asbestos modulates thioredoxin-thioredoxin interacting protein interaction to regulate inflammasome activation. *Part. Fibre Toxicol.* 11 1–13. 10.1186/1743-8977-11-24 24885895 PMC4055279

[B26] Warnatsch A. Ioannou M. Wang Q. Papayannopoulos V. (2015). Inflammation. Neutrophil extracellular traps license macrophages for cytokine production in atherosclerosis. *Science* 349 316–320. 10.1126/science.aaa8064 26185250 PMC4854322

[B27] Williams K. J. Tabas I. (1995). The response-to-retention hypothesis of early atherogenesis. *Arterioscler. Thromb. Vasc. Biol.* 15 551–561. 10.1161/01.ATV.15.5.551 7749869 PMC2924812

[B28] Yao Y. Mao J. Xu S. Zhao L. Long L. Chen L. (2018). Rosmarinic acid inhibits nicotine-induced C-reactive protein generation by inhibiting NLRP3 inflammasome activation in smooth muscle cells. *J. Cell. Physiol.* 234 1758–1767. 10.1002/jcp.27046 30146678

[B29] Zwaka T. P. Hombach V. Torzewski J. (2001). C-reactive protein-mediated low density lipoprotein uptake by macrophages: implications for atherosclerosis. *Circulation* 103 1194–1197. 10.1161/01.CIR.103.9.1194 11238260

